# Cytokines Adsorption During Ex Situ Machine Perfusion of Liver Grafts from Elderly Donors: A Pilot, Prospective, Randomized Study

**DOI:** 10.3390/life16010167

**Published:** 2026-01-20

**Authors:** Giulia Cirillo, Lorenzo Bernardi, Daniele Pezzati, Maria Franzini, Emanuele Balzano, Giovanni Tincani, Jessica Bronzoni, Caterina Martinelli, Arianna Trizzino, Lorenzo Petagna, Paola Carrai, Stefania Petruccelli, Ranka Vukotic, Erlis Uruci, Matilde Masini, Serena Babboni, Serena Del Turco, Riccardo Morganti, Vincenzo De Tata, Aldo Paolicchi, Giandomenico Biancofiore, Adriano Peris, Chiara Lazzeri, Giuseppina Basta, Davide Ghinolfi

**Affiliations:** 1 Division of Hepato-Biliary Surgery and Liver Transplantation, Azienda Ospedaliera Universitaria Pisana (AOUP), 56121 Pisa, Italyp.carrai@ao-pisa.toscana.it (P.C.); ranka.vukotic@ao-pisa.toscana.it (R.V.);; 2 Department of Surgery, Lausanne University Hospital (CHUV), University of Lausanne, 1011 Lausanne, Switzerland; lore.bernardi91@gmail.com; 3 Department of Translational Research and New Technologies in Medicine and Surgery, University of Pisa Hospital, 56121 Pisa, Italy; m.franzini@ao-pisa.toscana.it (M.F.);; 4 Institute of Clinical Physiology, National Research Council, 56124 Pisa, Italy; serenababboni@cnr.it (S.B.);; 5 Department of Statistical Support to Clinical Study, Azienda Ospedaliera Universitaria Pisana (AOUP), University of Pisa, 56121 Pisa, Italy; 6 Division of Anesthesia, Azienda Ospedaliera Universitaria Pisana (AOUP), University of Pisa, 56121 Pisa, Italy; g.biancofiore@ao-pisa.toscana.it; 7 Regional Transplant Authority of Tuscany (OTT), 50139 Florence, Italy

**Keywords:** cytokines, machine perfusion, cytokine adsorption, liver transplantation, ischemia–reperfusion injury

## Abstract

Ischemia–reperfusion injury (IRI) is a mechanism based on inflammatory mediators’ release and activation of effectors of damage. Studies showed a correlation between cytokine, severity of damage, and post-operative outcomes. Ex situ perfusion may work as a platform for the treatment of IRI mechanisms, such as the removal of cytokines using cytokine adsorption (CA). We assessed the safety and benefits of an integrated CA during ex situ dual-oxygenated hypothermic (D-HOPE) and normothermic perfusion (NMP). During the period of July 2021–December 2023, 84 octogenarian liver grafts, suitable for transplantation, were considered: 12 were randomized to D-HOPE or NMP with or without CA (D-HOPE + CA, D-HOPE, NMP + CA, NMP groups, n = 3 each) and compared to 72 performed using grafts preserved in static cold storage (SCS). IL-1, IL-6, IL-10, and TNF-a perfusate concentrations were evaluated together with perfusion parameters and post-operative outcomes. Perfusion procedures were unaffected by CA integration. In NMP, cytokine levels were 10–40 times higher than in healthy subjects and 20–50 times higher than D-HOPE. Cytokines were removed both in D-HOPE and NMP, but the concentration-dependent mechanisms of action of CA led to more remarkable removal in NMP. IL-10 and TNF-a concentrations were significantly lower in NMP + CA than in NMP. The application of CA was associated with significantly higher arterial flows both in D-HOPE and NMP, and reduced neutrophil infiltration in NMP. No differences in post-operative outcomes were found among groups. In conclusion, cytokine adsorption during ex situ machine perfusion of liver grafts from elderly donors is safe and feasible and is associated with modulation of inflammatory mediators and perfusion dynamics. These findings are hypothesis-generating, and larger studies are required to determine the clinical impact of this strategy.

## 1. Introduction

The use of elderly donors has the potential to expand the donor pool, reduce waiting list mortality, and broaden the clinical indications for liver transplantation (LT) [1]. Although several series have reported the successful use of grafts from elderly donors, these organs are often criticized due to their heightened susceptibility to post-LT complications, largely driven by their increased sensitivity to severe IRI [2,3,4].

IRI is a complex, multifactorial process resulting from depletion of glycogen and ATP due to the shift from aerobic to anaerobic metabolism. This metabolic disturbance causes cellular edema and dysfunction, and upon reperfusion results in increased mitochondrial membrane permeability, the release of reactive oxygen species (ROS), and activation of the pro-inflammatory cascade and cytokine release [5]. Cytokines are soluble, “hormone-like” mediators involved in response to stress and play critical roles in hemodynamic instability, organ injury, inflammation, and cell proliferation [6,7,8]. The liver is a source of cytokines: non-parenchymal liver cell populations, including lymphocytes, Kupffer, dendritic, and sinusoidal endothelial cells, can amplify the release of inflammatory mediators upon activation. While this immune response is essential for tissue healing, its uncontrolled or prolonged activation may result in graft injury and distant organ dysfunction [9].

Ex situ liver machine perfusion (MP) has been introduced to improve graft preservation and viability prior to LT. Studies showed that MP enables prolonged organ preservation, reduces IRI, and lowers the incidence of early graft dysfunction [6,10,11,12,13]. However, the immunological mechanisms underlying these benefits remain incompletely understood, particularly regarding the cytokines and immune profile shift that occur during perfusion. Some groups suggested that inflammatory cytokines and damage-associated molecular patterns (DAMPs) accumulate in the perfusate during MP, exacerbating the inflammatory response [14]. Some studies have also shown that MP can reduce inflammation by lowering neutrophil counts and promoting a shift from pro-inflammatory to aged or exhausted phenotype [14]. In this contest, perfusion fluid purification strategies such as CA have emerged as promising approaches to attenuate inflammation during MP [15]. CA uses hemoadsorption devices with porous polymer beads that selectively bind and remove inflammatory cytokines (typically 5–60 kDa), aiming to modulate immune activation, mitigate IRI, and enhance graft recovery. Experimental models have demonstrated that CA effectively reduces IL-6, TNF-a, IL-8, and DAMPs in various settings [16,17]. In particular, promising results have been reported in ex situ perfusion of other solid organs. In porcine DCD heart models, CA reduced cytokine levels, improved myocardial contractility, and limited tissue necrosis [18]. Similarly, in lung EVLP models, CA led to better oxygenation, improved compliance, and reduced systemic cytokine burden after transplantation [19,20]. These findings highlight the immunomodulatory potential of CA across the organ system. We have recently performed a preliminary evaluation on the application of CA in liver normothermic MP (NMP) in an animal model of donation after circulatory death (DCD) [21]. In similar experimental settings, other groups have explored CA in hypothermic MP (HMP), showing a potential role in reducing the inflammatory response after a severe ischemic injury, even in this downregulated metabolic scenario [22].

Therefore, the aim of this pilot prospective randomized study was to evaluate the safety and feasibility of integrating cytokine adsorption during ex situ liver machine perfusion of grafts fromelderly deceased donor after brain death (DBD) using both D-HOPE and NMP. We further sought to explore the biological effects of cytokine adsorption on inflammatory mediator dynamics, perfusion parameters, and early graft-related surrogate markers. We hypothesized that cytokine adsorption could be safely implemented during ex situ perfusion and would be associated with attenuation of inflammatory cytokine levels and improved perfusion characteristics, with potentially different effects between hypothermic and normothermic conditions.

## 2. Materials and Methods

### 2.1. Study Design and Setting

**Figure A1:**
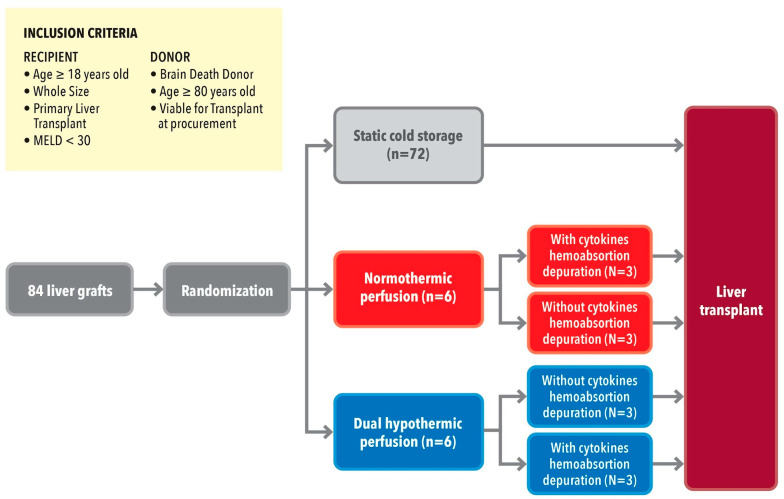
Study design.

This is a prospective, randomized, non-blinded study including patients who underwent LT using elderly (≥80 years) DBD donors at a single center. For the purpose of the study, all LTs performed using an octogenarian donor in the period from July 2021 to January 2023 were considered. During this period, 12 recipients were randomized to receive a liver graft preserved with dual hypothermic oxygenated perfusion (D-HOPE) or NMP (6 D-HOPE vs. 6 NMP) in the 2 MP treatment groups; a further randomization was performed to assign the grafts to CA or NO-CA (3 with CA vs. 3 NO-CA). Once a donor liver was deemed suitable for transplantation, the graft was assigned to a treatment group using a computer-generated randomization sequence, prepared in advance by an independent statistician not involved in patient care. Assignments were sealed in opaque, sequentially numbered envelopes. The study team opened the envelope immediately before starting ex situ perfusion to determine whether the graft would undergo D-HOPE or NMP, and, within each MP group, whether CA would be applied. This process ensured allocation concealment and prevented selection bias. The clinical team performing transplantation was aware of the perfusion modality due to procedural requirements, but the envelope assignment was independent and blinded until perfusion initiation. MP was never used for organ viability assessment, and grafts were clinically used regardless of flows and perfusate metabolites (e.g., lactate, glucose, or pH) or bile production, as all grafts perfused ex situ were considered transplantable before perfusion. After the first twelve donors randomized to MP, all subsequent octogenarian DBD grafts were preserved using SCS and were includedfor comparison until a ratio of 1:6 (n = 72) was reached. A minimum of 2 years of follow-up was required. Study design is reported in Appendix A (Figure A1).

The sample size case (3 per group) was required to have an 80% chance of detecting, significant at the 5% level, a decrease of 40% of the IL-10 concentration from an estimated 100 mcg/mL in the experimental group. This means that with the current sample size (n = 12), the study is sufficiently powered to only detect interleukin concentration and provide safety and feasibility of using CA during organ perfusion, but is not powered to investigate clinical outcomes. It was conducted in compliance with the CONSORT guidelines for prospective randomized studies. The study protocol was approved by the Institutional Review Board of the University Hospital of Pisa, Italy, as part of the DCDNet study (CEAVNO, protocol#17484). The recipients provided written informed consent to the current analysis. All the research was conducted according to the Helsinki and Istanbul declarations.

### 2.2. Participants (Recipient and Donor Selection)

All adult recipients (≥18 years old), eligible for primary whole-size LT, with MELD < 30, were considered for the study. Consecutive DBD donors ≥ 80 years old with viable organs for LT were included.

### 2.3. Allocation and Transplantation Criteria

Eligibility for liver donation was evaluated as per our institutional policy and according to the Italian National Transplant Agency (Centro Nazionale Trapianti [CNT]) guidelines. The evaluation policy for octogenarian donors is based on center-specific and national guidelines [23,24,25].

### 2.4. Liver Procurement

The liver procurement was carried out according to our standard *en-bloc* institutional technique. Liver graft biopsy was performed on demand based on surgical evaluation, and grafts were discarded in the presence of macro-vesicular steatosis > 40%, fibrosis > 2 as per Ishak’s score, and macro-angiopathy precluding arterial anastomosis [25]. All grafts were routinely evaluated on the back table before LT for vessel patency and anatomical variants.

### 2.5. Ex Situ Organ Preservation

#### 2.5.1. SCS Procedures

In the SCS arm, the organ retrieval, storage, and transplants were conducted according to standard practice.

#### 2.5.2. MP Procedures

Both HMP and NMP were performed end-ischemically in an operating room (OR) next to the transplant OR under medical supervision as described elsewhere using the PerLife^®^ device (Aferetica, Bologna, Italy) in PerLiver operational mode [25]. The duration of perfusion was 2 h for DHOPE and 4 h for NMP when feasible, as per our standard protocols. 

### 2.6. Purification of the Perfusate by Sorbent Device During MP

The MP circuit was integrated with a CA cartridge (PerSorb^®^, Cytosorbents Inc., Princeton, NJ, USA) during ex situ perfusion. This device contains a highly porous polymer adsorbent capable of removing hydrophobic molecules up to approximately 60 kDa, including cytokines, bilirubin, and myoglobin, for up to 24 h. The removal mechanism is non-specific and concentration-dependent, meaning that higher concentrations of target molecules are more readily adsorbed, while lower levels are less affected. This dynamic enables partial cytokine clearance, which may help mitigate excessive inflammation without complete depletion of key mediators. However, the device does not allow selective targeting and restoration of physiological cytokine levels, and its effect on immune modulation remains indirect and variable. The CA device was placed in a parallel circuit connected via a dedicated roller pump, allowing a maximum flow of 500 mL/min through the cartridge, as previously described [25]. CA is intended as an adjunct immunomodulatory strategy to reduce excessive inflammatory mediators during ex situ perfusion and potentially mitigate ischemia–reperfusion injury. It is not a selective cytokine therapy and does not replace standard perfusion protocols.

### 2.7. LT Surgery

All transplants were performed using either the conventional piggy-back technique or with vena cava replacement and venous–venous bypass. A T-tube was eventually used for duct-to-duct biliary anastomosis according to surgeon preference; the tube was removed three months post-transplantation after trans T-tube cholangiography.

### 2.8. Data Collection

#### 2.8.1. Donor, Graft, and Recipient

Donor, recipient, and graft characteristics were gathered from the prospectively maintained institutional database. The recipient’s model for end-stage liver disease (MELD) score was calculated as laboratory MELD without the inclusion of exception points for HCC.

Donor and recipient demographics and clinical data were collected to characterize the donor–recipient matching and clinical outcomes of the transplantation (Table 1).

Graft procurement and transplantation are characterized in terms of cold (CIT) and warm (WIT) ischemia time, extraction and transplantation time, vascular reconstructions, biopsies, and graft weight.

#### 2.8.2. MP Characteristics

During MP treatments, hemodynamic, metabolic, and synthetic parameters were recorded hourly and monitored from the beginning until the end of the treatment: hepatic artery and portal vein pressures and flows, temperature, perfusate glucose, transaminases, and lactates. In NMP treatments, bile production and quality were assessed.

#### 2.8.3. Cytokine Levels Determination

Perfusate was collected every hour and analyzed for cytokine (interleukin-1 (IL-1), interleukin-6 (IL-6), interleukin-10 (IL-10), and tumor necrosis factor α (TNFα)level characterization with ELISA. The panel of cytokines selected for analysis was chosen based on their well-established roles in the inflammatory cascade associated with IRI and their relevance in organ preservation and transplantation outcomes; IL-1 and TNFα are early pro-inflammatory cytokines released by activated immune and endothelial cells in response to tissue injury. They act as upstream mediators of the innate immune response, amplifying leukocyte recruitment, endothelial activation, and further cytokine release. IL-6 is a central regulator of acute-phase inflammation and is consistently associated with the severity of IRI and early graft dysfunction in both preclinical and clinical transplantation studies. It also reflects hepatocellular stress and contributes to systemic inflammation. IL-10 serves as a key anti-inflammatory cytokine, counterbalancing the pro-inflammatory response. It is important in limiting immune-mediated tissue damage and promoting immune tolerance. Its levels provide insight into the graft’s ability to regulate inflammation. Together, this cytokine panel allows for a comprehensive assessment of the balance between pro- and anti-inflammatory signals during machine perfusion and after transplantation. Monitoring their dynamics in the presence or absence of CA provides mechanistic insight into the immunomodulatory effects of CA and helps evaluate its potential to reduce inflammation-related graft injury. Other mediators, such as DAMPs, were not included in this initial clinical study due to technical limitations and the need to focus on well-characterized cytokines with established assays, allowing for reproducible and interpretable results. Future studies may expand the panel to include DAMPs and other inflammatory mediators to provide a more comprehensive understanding of graft immunobiology.

#### 2.8.4. Liver Biopsies

Liver graft biopsies were taken at three pre-defined time frames: end of the back table (t_1_), end of MP (t_2_), end of LT (t_3_), and evaluated using hematoxylin and eosin (H&E) and PAS coloration. Electronic microscopy (EM) evaluation was also performed as described elsewhere [26,27].

All perfusate and tissue analyses were conducted according to standard protocols qualified and validated for the scope within our facilities.

#### 2.8.5. Variables, Clinical Outcomes, and Definitions

Outcomes. The characterization of the feasibility, safety, and efficacy of the perfusion liquid purification with CAD was performed considering the following.

(a) Perfusate cytokine levels and CA efficacy determination with the mass balance (MB) calculation are expressed as follows.

The mass balance (MB) during the time period (t_2__-__1_) was measured by evaluating cytokine concentration before and after CA with the following formula:

(C_pre_ − C_post_) ∗ flow ∗ time

where C_pre_ = (C_t1_ + C_t2_)/2), and C_post_ = (C_t1_ + C_t2_)/2).

MB can be used to describe the dynamics involving the target molecule’s release/generation and removal in the organ, allowing the evaluation of interventions like adsorption. It offers the comparison of the levels of the target molecule in the sorbent’s input and output to assess not only the effectiveness of the purification process, but also the amount of inflammatory mediators’ release/production by the graft.

(b) The effect of CA on MP hemodynamics: Vascular flows were measured by the study group and normalized by liver weight as well.
(c) Effect of CAon liver graft histology and inflammation.

Histological and electron microscopic analyses were used to assess the graft injury and inflammation in the three study groups.

Lastly, an exploratory comparison among the study groups was performed to assess the clinical outcomes. The primary non-function (PNF) was defined as the graft failure with no identifiable secondary cause resulting in either re-LT or death within the first week. The early allograft dysfunction (EAD) was defined as per Olthoff et al. [28]. Post-reperfusion syndrome (PRS) was defined as a decrease in mean arterial pressure (MAP) greater than 30% below the baseline value, lasting for at least 1 min, and occurring during the first 5 min after reperfusion of the liver graft [29]. Acute kidney injury (AKI) was defined according to KDIGO guidelines [30]. Renal rescue therapy (RRT) was adopted when indicated. Post-LT complications were graded according to the Clavien–Dindo classification and the comprehensive complication index (CCI) [31]. Among vascular and biliary complications, the incidence of ischemic cholangiopathy (IC), anastomotic stenosis (AS), and biliary leakage was recorded. The IC was defined as diffuse intrahepatic, hilar, or extrahepatic biliary strictures requiring treatment in the presence of a patent hepatic artery. Biliary leakage was defined according to the International Study Group for Liver Surgery (ISGLS) [32]. Finally, graft and patient survival were estimated in each group.

### 2.9. Statistical Analysis

According to their level of measurement and distribution, continuous variables were expressed as means and standard deviations (SDs) or medians and interquartile ranges (IQRs), while categorical variables were described as frequencies. Data were compared with the *t*-test or ANOVA for continuous values with normal distribution, the Mann–Whitney U test for continuous values without normal distribution, and Pearson’s chi-square or Fisher’s exact tests for categorical values. To account for multiple statistical comparisons, *p*-values were adjusted using the Bonferroni correction where appropriate. The level of significance was set at 5%. Survival was estimated by the Kaplan–Meier method. All analyses were carried out by SPSS v.28 technology.

## 3. Results

During the study period, 84 patients underwent LT using liver grafts from octogenarian DBD donors. Six patients underwent LT after organ preservation with NMP and six after D-HOPE. In each of the two groups, three patients were randomized to CA. Seventy-two patients underwent LT after standard SCS graft preservation. Donor, recipient, and transplant characteristics are summarized in Table 1 and Table 2.

### 3.1. Cytokines Concentration During Ex Situ Perfusion and Efficacy of CA

**Figure 1:**
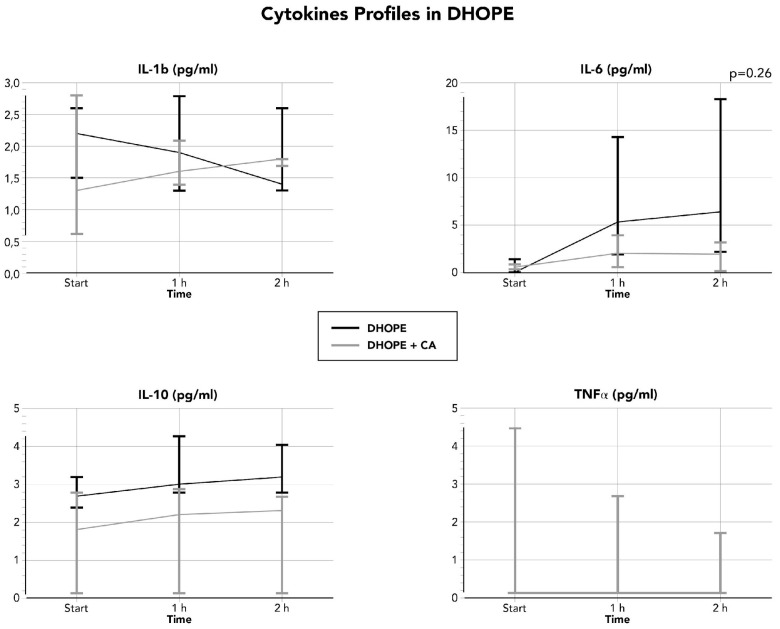
Perfusate cytokine concentration during D-HOPE.

**Figure 2:**
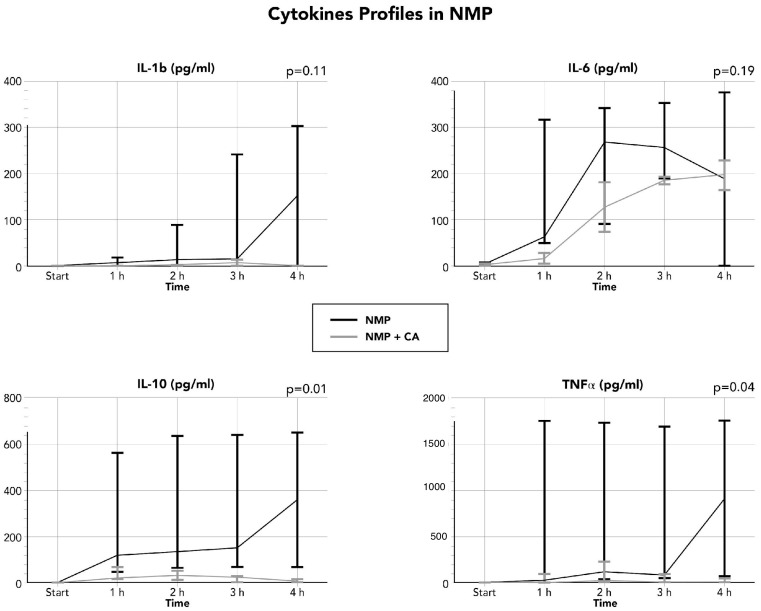
Perfusate cytokine concentration during NMP.

Overall cytokine perfusate concentration is reported in Table 3, while detailed concentration stratified by type of MP and the use or not of CA is in Figure 1 (D-HOPE) and **2** (NMP). During D-HOPE, the concentration of cytokines (IL-1, IL-6, IL-10, and TNFa) is lower than NMP; moreover, the use of CA did not provide any major differences at lower temperatures. Only perfusate IL-6 showed a slight trend (not statistically significant) towards a higher concentration when CA was not used (*p* = 0.26). On the contrary, NMP was characterized by a higher concentration of cytokines, and CA significantly reduced TNF-a (*p* = 0.04) and IL-10 (*p* = 0.01), while other cytokines showed a non-significant trend (Table 3 and Figure 1 and Figure 2).

The mass balance during D-HOPE and NMP is reported in Table 3. During the first two hours of perfusion, a mean amount of 2520 and 5124, 6720 and 231.327, 0 and 177.276, 0 and 12.738 pg of IL-1, IL-6, IL-10, and TNF-a were removed in D-HOPE and NMP, respectively. During 4 h of NMP, a total amount of 9.075, 792.306, 436.689, and 192.048 pg of IL-1, IL-6, IL-10, and TNF-a were removed, respectively.

### 3.2. Effect of the Cytokines CA on the Hepatic Artery and Portal Vein Flows

**Figure 3:**
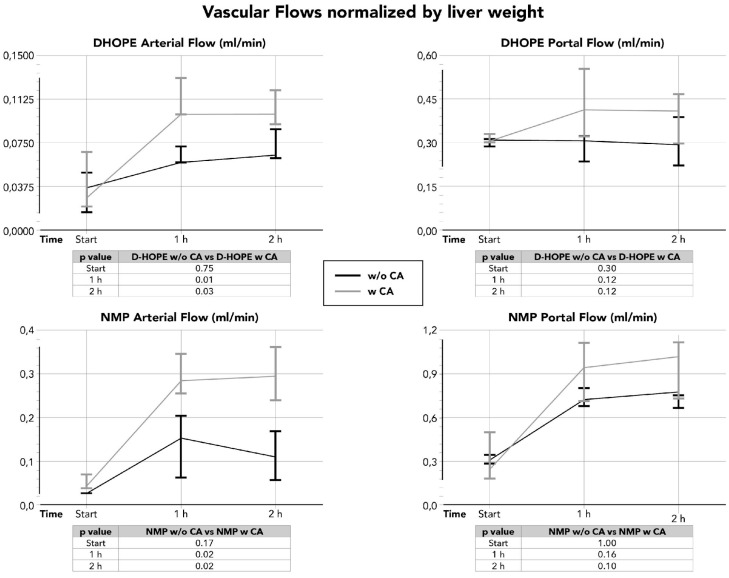
Vascular flows in hepatic artery and portal vein during D-HOPE and NMP normalized by liver weight.

**Figure A2:**
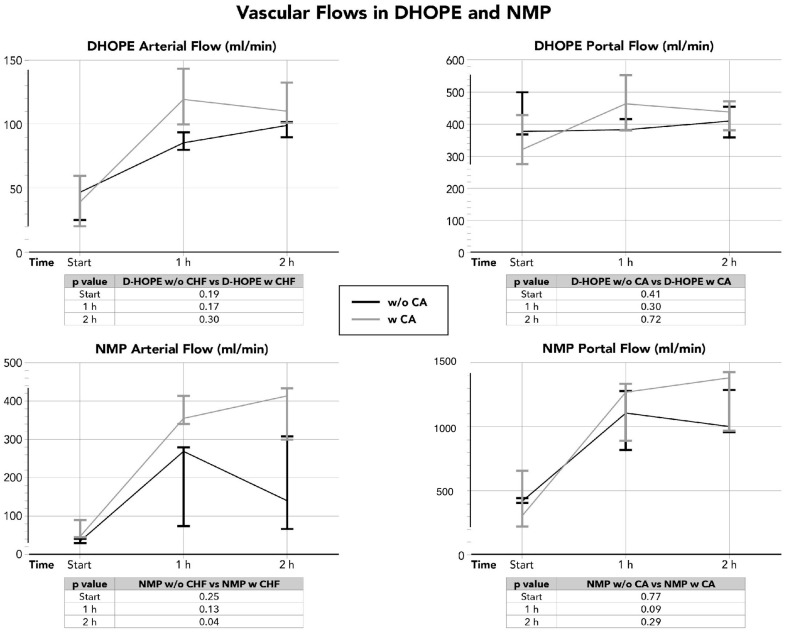
Vascular flows in hepatic artery and portal vein during D-HOPE and NMP.

Vascular flows in both the hepatic artery and portal vein during D-HOPE were similar at the beginning of MP and after 1 and 2 h of MP, irrespective of the use of CA (Figure A2), but were significantly higher in the hepatic artery if normalized by liver weight (Figure 3).

Vascular flow in the hepatic artery at the beginning of NMP was similar, irrespective of the use of CA. In those grafts perfused with the CA, the arterial vascular flow increased after 1 h and became significantly higher at 2 h after the beginning of NMP. When normalized by liver weight, arterial flows were higher at 1 and 2 h when a CA was used (Figure 3).

### 3.3. Histology Evaluation of Liver Graft Biopsies

Histology, together with Suzuki’s score, is summarized in Table A1. Even if a trend towards worse damage was present in the NMP w/o CA group, no significant differences were shown.

### 3.4. Electronic Microscopy Evaluation (EM) of Liver Graft Biopsies

**Figure 4:**
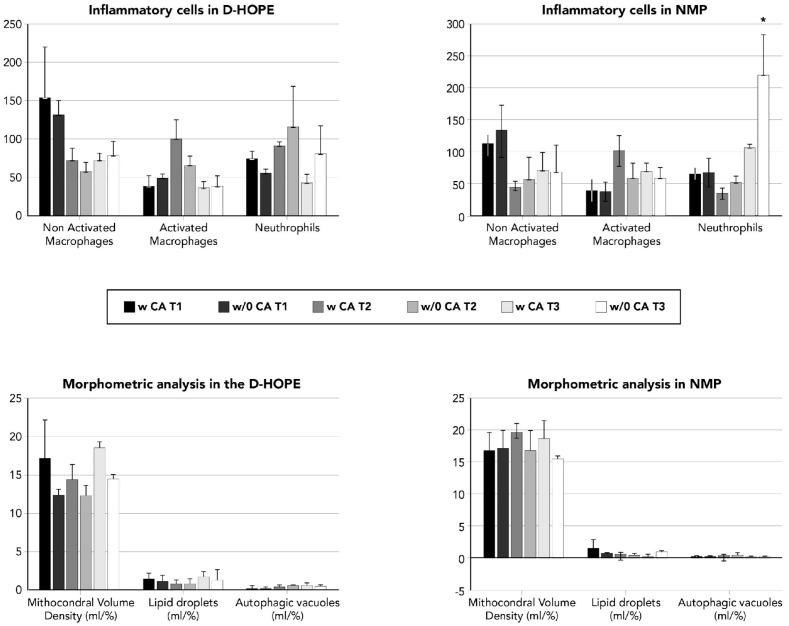
Electron microscopy evaluation of liver graft biopsies required a median of 1 procedure, where 1 is still on treatment after 4 procedures, and 2 required re-LT. * means statistically relevant.

In liver biopsies taken on the back table during MP and at the end of LT, the neutrophil infiltration was similar irrespective of the use of CA in D-HOPE but significantly increased during NMP at the end of LT when a CA was not used (*p* = 0.05) (Figure 4).

### 3.5. Overall Clinical Outcomes

The clinical outcomes in the three groups are summarized in Table 1. Even if there were no significant differences in clinical outcomes, a trend towards lower post-LT transaminases was shown in the D-HOPE group. Patients whose grafts were preserved with MP showed a trend towards better graft and patient survival. The NMP group required an early re-transplantation for a case of severe post-reperfusion syndrome, while the reasons for graft loss in the SCS group within 3 months after LT were sepsis and multiorgan failure (n = 7), hepatic artery thrombosis (n = 3), and an incidental donor cancer finding with the following liver explant in one case.

There was no difference in terms of biliary complications: SCS showed eight cases of IC (11%), as well as D-HOPE and NMP with one case each, and with an onset at a median time of 185 (59–242), 315, and 560 days, respectively. All cases of IC in the MP-treated grafts were amenable to ERCP treatment after three procedures in the D-HOPE, one in the NMP, and five cases in the SCS.

### 3.6. Clinical Outcomes (D-HOPE and NMP, with and Without CS)

The clinical characteristics of patients who underwent LT with grafts perfused by D-HOPE and NMP with or without additional CA purification are summarized in Table 2. No major differences were found in the clinical outcomes among groups. One case in the NMP group without CA required re-LT because of a severe PRS and recipient instability. All biliary complications of the D-HOPE group happened without CA, while the IC in the NMP group used CA. No other major event occurred in the remaining cases.

TNF-a showed a significant variability between groups (*p* = 0.031) and days (*p* = 0.017), showing a tendency to decrease in time and lower value when CA was used. IL-6 and IL-10 showed a variability between groups (*p* = 0.006 and *p* = 0.054, respectively).

Bile characteristics are reported in Appendix B (Table A2). All patients showed alkalotic bile, with low glucose and lactate since the first post-operative day, with no major differences between the study group and no association with the future development of IC. Only the patient who showed the severe PRS had higher lactate levels.

## 4. Discussion

This paper reports the first clinical use of CA during liver ex situ MP, evaluating its potential to optimize liver graft preservation by modulating inflammatory mediator levels in the perfusate. The relationship between IRI and the inflammatory cascade during MP needs further exploration; cytokines and other inflammatory mediators play a pivotal role in triggering and amplifying IRI [10]. Our findings align with growing literature indicating a link between cytokine expression and clinical outcomes. The novelty of this study lies in the first demonstration that cytokine adsorption can be safely integrated into liver MP, modulate inflammatory mediators, and influence key cellular and hemodynamic parameters. Clinically, this suggests a new strategy to potentially optimize graft preservation, improve early function, and reduce IRI-related complications, a crucial advancement given the ongoing shortage of high-quality donor livers and the challenges of marginal grafts. For instance, we recently reported a correlation between post-liver transplant graft loss and perfusate IL-6 levels [33]. Similarly, Ly et al. [34] identified IL-6 in bile as a key marker of liver regeneration during prolonged NMP. In kidney and lung MP models, preliminary clinical experiences with CA have demonstrated favorable safety profiles and suggested potential benefit in post-transplant function [35,36]. Moreover, our findings are broadly consistent with previous experimental work. Gruda et al. [17] showed that CA significantly reduced levels of multiple inflammatory mediators in whole blood in vitro, while Jansen et al. [16] demonstrated reduced systemic cytokine levels in healthy volunteers challenged with lipopolysaccharide following CA hemoperfusion. Obara et al. [15] explored early perfusate replacement in a murine liver transplant model and found that even non-adoptive clearance of inflammatory substances improved graft injury markers, underscoring the potential utility of cytokine removal during MP. In our work, we have characterized cytokine expression during D-HOPE and NMP and evaluated the effects of the application of a CA in MP. CA proved feasible and safe in all procedures, with no adverse effects or interference with graft perfusion, and no need to adjust perfusate volume.

D-HOPE showed 10 to 20 times lower levels of cytokines than NMP. Even so, both in D-HOPE and NMP, IL-6, IL-10, IL-1, and TNF-a are overabundant in comparison to the total serum healthy subjects’ cytokine levels, suggesting an important inflammatory response of the graft during the treatment [37,38]. The effects of this huge and prolonged exposure of a liver graft to several inflammatory mediators have to be investigated and understood. Notably, CA appeared more effective in NMP than D-HOPE, likely due to the higher baseline cytokine concentrations in NMP, which provide a greater substrate for adsorption. In addition, the higher perfusion temperature and metabolic activity during NMP may amplify cytokine production, making removal via CA more impactful. This mechanistic insight highlights the potential for temperature- and metabolism-dependent modulation of inflammatory mediators during MP.

Even if the small number of cases performed does not allow for drawing any final clinical conclusion, the use of CA has two major effects. The first is the capacity of the device to improve arterial perfusion of the graft, either when these are normalized or not by liver weight. Even if there are no definitive data that an increased arterial flow is associated with better clinical outcomes, the grafts formed a more physiological relationship between arterial and portal flow.

The second is at a microscopic level: no differences could be found in terms of histological damage or neutrophil recruitment during D-HOPE, while a significant difference was shown in liver grafts perfused in NMP ex situ. Neutrophils are the most important effectors of the inflammatory response during IRI, and their reduced presence when cytokine perfusate levels are minimized by CA could be an indirect demonstration that controlling cytokine concentration may lead to a reduced IRI.

From a clinical point of view, the limited number of cases does not allow for capturing major differences among groups; nevertheless, a trend towards a lower transaminases peak after LT in the D-HOPE group is shown, even if with no influence on hospital stay or graft/patient survival. Interestingly, the SCS group develops IC earlier than those grafts that undergo MP, justifying a prolonged follow-up for an adequate assessment of post-LT complications. Importantly, the lack of observable differences in clinical outcomes does not negate the biological impact of CA, as we documented clear microscopic and perfusate-level effects, particularly in NMP. These results indicate that while immediate clinical endpoints may remain unchanged in a small series, CA may still exert early protective effects at the cellular and vascular level, which could translate into clinical benefits in larger studies. Therefore, our findings highlight the need for well-powered trials to clarify the true clinical significance of cytokine adsorption during liver machine perfusion.

Our experience also has several limitations: firstly, the limited number of cases, which decreases the strength of our conclusions, and secondly, the limited number of cytokines evaluated. Additionally, the study was not powered to assess clinical endpoints, and only a limited panel of cytokines was evaluated, leaving other inflammatory mediators and potential mechanisms unexplored. Moreover, it was conducted at a single center, which may affect the generalizability of the findings. It is worthwhile to mention that the control of perfusate cytokine concentration can be achieved in several other ways, such as the full change in the perfusate, the use of a lower temperature, the minimization of ischemic times (e.g., IFOT technique), or by treating the donors or the liver grafts during ex situ perfusion pharmacologically or with nanotechnology [37,38], even if, up to date, the adsorption is the only one approved for clinical use.

In conclusion, cytokine perfusate concentration during MP in response to ischemic damage changes depending on the temperature of reperfusion. The levels of cytokines can be modulated by the use of CA with unselective and concentration-dependent mechanisms without negatively affecting them in comparison to the physiological values or MP safety. CA promotes a decrease in the concentration of neutrophils in the grafts, which are the effectors of inflammatory response, improving vascular flows and may have a role in containing the inflammatory response/activation during perfusion. The clinical impact of cytokine adsorption in MP must be furtherly validated in rigorous trials.

## 5. Conclusions

In conclusion, CA during ex situ liver machine perfusion is feasible and safe. CA effectively reduces inflammatory cytokine levels, improves vascular perfusion, and decreases neutrophil infiltration in the graft, suggesting a potential role in mitigating ischemia–reperfusion injury. While these findings are encouraging, larger studies are required to confirm the clinical benefits of CA in liver transplantation.

## Figures and Tables

**Table 1:** Donor, recipient, and transplant characteristics and outcomes stratified by type of organ preservation.

Variable	SCS (n = 72)	NMP (n = 6)	D-HOPE (n = 6)	*p* ^SCS-NMP^	*p* ^SCS-DHOPE^	*p* ^NMP-HOPE^
**Donor Characteristics**						
Age, years	84 (81.8–87)	82.5 (81.3–84.5)	82 (81.3–86.5)	0.251	0.641	0.547
Gender (M/F)	44/28 (61/39)	3/3 (50)/(50)	2/4 (33)/(67)	0.599	0.188	0.599
Location—regional	6 (100)	6 (100)	6 (100)	1.0	1.0	1.0
Weight, Kg	70 (60–80)	68.5 (65–74.3)	75 (63.8–85.5)	0.556	0.731	0.575
Height, cm	170 (164–175)	174 (167–175)	168 (162–173)	0.341	0.748	0.384
BMI	24.5 (23–27)	24 (21–24)	25–5 (23.3–28.5)	0.120	0.566	0.207
Cause of death:						
CVA	60 (83)	5 (83)	5 (83)	0.887	0.887	1
Trauma	10 (14)	1 (17)	1 (17)	-	-	-
Anoxia	2 (3)	0	0	-	-	-
ICU stay (days)	3 (2–5.25)	2 (2–2)	2 (2–2.75)	0.153	0.151	1
Comorbidities:						
Hypertension	58 (81)	2 (33)	1 (16)	0.008 *	0.239	0.01 *
Diabetes	16 (22)	1 (16)	1 (16)	0.755	0.755	1
Cardiomyopathy	44 (61)	2 (33)	3 (50)	0.188	0.599	0.599
Dislipidemy	29 (70)	3 (50)	4 (67)	0.647	0.214	0.599
Nephropathy	8 (11)	1 (16)	1 (16)	0.687	0.687	1
Virology:						
Hbc/HBV DNA+	11/1 (15/1.3)	1 (16)	1 (16)	0.929/0.775	0.929/0.775	1/-
HCVAb/HCV RNA	1/0 (1.3/0)	0/0	0/0	0.775	0.775	-
Last lab values:						
Na, mEq/L	148 (145–153)	147 (145–152)	146 (141–149)	0.963	0.204	0.191
AST, UI/L	24 (19–37.5)	20.5 (15–36.5)	19.5 (17.3–39)	0.907	0.614	0.583
ALT, UI/L	17 (11–29)	12 (10–23)	10 (8–17.3)	0.491	0.323	0.342
Bilirubin, mg/dL	0.6 (0.42–1.02)	0.395 (0.32–0.7)	0.68 (0.39–0.7)	0.290	0.483	0.659
**Recipient Characteristics**						
Age, years	58 (54–63)	62 (58.5–64.8)	54.5(53.3–58.8)	0.758	0.76	0.66
Gender (M/F)	49/23 (68/32)	4/2 (67/33)	4/2 (67/33)	0.945	0.945	1
Weight, Kg	72 (62–76.5)	74 (72.3–84.8)	75 (70.5–91.5)	0.130	0.246	0.812
Height, cm	168 (160–173)	173 (170–177)	169 (168–175)	0.309	0.331	0.295
BMI	25 (22–28)	25 (22.8–28)	26 (25–29.3)	0.528	0.403	0.907
MELD	11.5 (9–15)	12.5 (9–13)	11.5 (8.5–13.8)	0.960	0.632	0.717
Cause of transplant:						
HCC	32 (44)	2 (33)	2 (33)	0.525	0.953	0.563
HCV	4 (5.5)	0	0	-	-	-
HBV	9 (12.5)	0	1 (16)	-	-	-
NASH	5 (7)	1 (16)	2 (33)	-	-	-
ETOH	9 (12.5)	2 (33)	1 (16)	-	-	-
CSP/CBP	4 (5.5)	1 (16)	0	-	-	-
Other	9 (12.5)	0	0	-	-	-
**Procurement Characteristics**						
Extraction time, min	23.5 (14–30)	22 (12.5–31.5)	10 (10–17.5)	0.832	0.085	0.203
Arterial reconstruction	12 (16)	0	1 (16)	0.283	1	0.341
Biopsy (Y/N)	33 (45)/39 (55)	2 (33)/4 (67)	1 (16)/5 (84)	0.560	0.171	0.549
CIT, min	450 (392–488)	292 (274–306)	248 (237–259)	<0.01 *	<0.01 *	0.04 *
Re-cooling time, min	-	28.5 (18.3–19.5)	17.5 (17–19.5)	-	-	0.113
WIT, min	45 (37–55)	46 (33.3–61.8)	49.5 (30–54)	0.858	0.648	0.851
Liver weight, gr	1320 (1180–1408)	1263 (1203–1491)	1250 (1040–1393)	0.908	0.436	0.432
Liver weight post-MP, gr	-	1236 (1196–1403)	1298 (1055–1465)	-	-	0.589
**Transplant Characteristics**						
Duration, min	365 (320–420)	362 (340–426)	349 (324–372)	0.591	0.386	0.308
Cava replacement (yes)	48 (66)	3 (50)	4 (67)	0.416	1	0.599
Biliary reconstruction:						
c-c, N	70 (97)	6 (100)	6 (100)	0.351	0.351	1
e-d, N	2 (3)	0	0	0.684	0.684	-
**Outcomes**						
PNF (yes)	0 (0)	1 (17)	0 (0)	1.0	1.0	1.0
PRS (yes)	15 (21)	1 (17)	0 (0)	1.0	0.58	1.0
EAD (yes)	11 (15)	1 (17)	0 (0)	0.67	0.61	1.0
AKI (yes)	19 (26)	2 (34)	0 (0)	0.96	0.36	0.43
RRT (yes)	6 (8%)	1 (17%)	0 (0)	0.46	0.95	1.0
PRBC, units	2 (0–4)	2 (0–3.5)	2 (1–4)	0.70	0.53	1.0
FFP, units	3 (1–5)	3.5 (2–5.5)	2 (1–4)	0.76	0.12	0.32
AST peak, UI/L	461 (294–1042)	663 (477–1500)	240 (208–327)	0.55	0.23	0.06 *
ALT peak, UI/L	433 (277–720)	279 (243–1253)	135 (124–199)	0.44	0.11	0.07 *
Hospital stay, days	13 (10–23)	15 (10–20)	13.5 (9–20)	0.95	0.62	0.71
CD > 3°	8 (11)	1 (17)	0 (0)	0.55	1.0	1.0
CCI at discharge	20.9 (8.7–31.5)	21.1 (8.7–35.5)	8.7 (8.7–17.8)	0.97	0.77	0.70
Vascular complication (y)	5 (7)	0	0	0.508	0.508	-
Biliary complicat., tot	26 (36)	1 (16)	2 (33)	0.343	0.893	0.549
-ischemic cholangiopathy	8 (11)	1 (16)	1 (16)	0.687	0.687	-
-anastomotic stenosis	13 (18)	0	1 (16)	0.260	0.933	0.734
-fistula	3 (4)	0	0	0.616	0.616	-
-stones	2 (2.7)	0	0	0.684	0.684	-
1-year graft survival, %	78	84	100	0.755	0.200	0.341
1-year patient survival, %	85	100	100	0.308	0.308	-
Bil. at 1 year, mg/dL	0.99 (0.6–1.52)	1.15 (1–1.19)	0.55 (0.49–0.62)	0.7	0.630	0.086
GGT at 1 year, UI/L	49 (27–121)	34 (23–58)	22.5 (17–28)	0.426	0.161	0.441
Median follow-up (months)	29 (24.8–35)	32.5 (28.3–33)	38 (37.3–38)	0.473	0.031	0.006

Abbreviations: SCS: static cold storage; NMP: normothermic machine perfusion; D-HOPE: dual hypothermic machine perfusion; BMI: body mass index; CVA: cerebrovascular accident; ICU: intensive care unit; MELD: model for end-stage liver disease; CIT: cold ischemia time; WIT: warm ischemia time; PNF: primary non-function; PRS: post-reperfusion syndrome; EAD; early allograft disfunction; AKI: acute kidney injury; RRT: renal replace therapy; PRBC: packed red blood cell; FFP: fresh frozen plasma; CD: Clavien–Dindo; CCI: comprehensive comorbidity index. Data are reported in median and interquartile range or number and percentage as appropriate. * indicate statistically significant differences (*p* < 0.05). Bold characters in the table footer are used to denote the table’s subtitles.

**Table 2:** Donor, recipient, and transplant characteristics and outcomes stratified by use of cytokine hemadsorption filter.

Variable	D-HOPE w CAD	D-HOPE w/o CAD	*p*	NMP w CAD	NMP w/o CAD	*p*
**Donor Characteristics**						
Age, years	88 (85–89)	81 (81–82)	0.09	83 (82–84)	81 (80–84)	0.65
Gender (M/F)	1/2 (33/66)	1/2 (33/66)	1.0	2/1 (66/33)	1/2 (33/66/)	1.0
Weight, Kg	60 (52–68)	89 (82–90)	0.07	65 (60–70)	72 (68–74)	0.43
Height, cm	160 (157–169)	168 (167–172)	0.45	165 (162–170)	175 (173–177)	0.15
BMI	23.4 (21.1–23.7)	29.0 (27.1–30.6)	0.04	24.5 (22.3–24.99)	24 (22.0–24.2)	0.80
Cause of death (CVA)	3 (100)	2 (66)	1.0	2 (66)	3 (100)	1.0
ICU stay (days)	2 (1.5–3.5)	2 (2–2.5)	0.80	2 (2–2)	2 (1.5–4)	0.55
Comorbidities:						
-diabetes	3 (100)	0	0.10	0	1 (33)	1.0
-hypertension	3 (100)	3 (100)	1.0	1 (33)	1 (33)	1.0
-cardiopathy	1 (33)	1 (33)	1.0	1 (33)	1 (33)	1.0
-dyslipidemia	2 (66)	1 (33)	1.0	2 (66)	1 (33)	1.0
-nephropathy	1 (33)	0	1.0	0	1 (33)	1.0
-anti HBc pos	1 (33)	0	1.0	0	1 (33)	1.0
Last lab values:						
-AST, UI/L	45 (29–46)	18 (17–20)	0.18	18 (15–30)	23 (18–65)	0.33
-ALT, UI/L	19 (12–19)	8 (8–10)	0.38	10 (8–18)	14 (12–28)	0.55
-BIL T, mg/dL	0.7 (0.5–0.8)	0.7 (0.5–0.8)	0.83	0.3 (0.3–0.4)	0.8 (0.6–1.0)	0.11
**Recipient characteristics**						
Age, years	55 (54–60)	54 (53–57)	0.67	65 (51–67)	60 (59–62)	0.72
Gender (M/F)	1/2 (3/66)	3/0 (100/0)	1.0	1/2 (3/66)	3/0 (100/0)	1.0
Weight, Kg	72 (65–84)	74 (71–87)	0.70	72 (68–92)	75 (74–81)	0.80
Height, cm	168 (166–174)	170 (169–173)	0.87	170 (170–174)	175 (172–180)	0.47
BMI, N	27.0 (23.8–28.5)	25.3 (25.1–29.1)	0.66	25.3 (23.5–27.1)	24.9 (23.5–30.3)	0.65
MELD	13 (11–15)	8 (7–11)	0.29	13 (12–13)	8 (7–15)	0.95
**Procurement Characteristics**						
Arterial reconstruction N	0	1	1.0	0	0	1.0
CIT, min	228 (218–251)	231 (227–238)	0.24	240 (238–260)	269 (266–294)	0.24
WIT, min	55 (39–62)	48 (36–50)	0.65	65 (48–70)	40 (30–46)	0.29
Liver weight, gr	980 (937–1205)	1280 (1250–1442)	0.26	1225 (1210–1262)	1555 (1367–1585)	0.21
Liver weight post-MP, g	1020 (978–1248)	1435 (1297–1532)	0.29	1260 (1225–1355)	1215 (1200–1552)	0.62
MP duration, min	128 (125–143)	157 (145–162)	0.34	279 (274–293)	241 (220–244)	0.04
**Transplant Characteristics**						
Cava replacement (yes)	2	2	1.0	1	2	1.0
Biliary reconstruction						
-duct to duct w T-tube	3	3	1.0	3	3	1.0
**Outcomes**						
PNF (yes)	0	0	1.0	0	1	1.0
PRS (yes)	0	0	1.0	0	1	1.0
EAD (yes)	0	0	1.0	0	0	1.0
AKI (yes)	0 (0)	0 (0)	1.0	1 (33)	1 (33)	1.0
RRT, (yes)	0 (0)	0 (0)	1.0	0 (0)	1 (33)	1.0
PRBC, N units	4 (3–5)	0 (0–1)	0.07	1 (0.5–2)	2 (1–9)	0.45
FFP, N units	2 (2–3)	1 (1–3)	0.51	4 (3–5)	3 (1–5)	0.90
AST peak, UI/L	260 (232–452)	220 (203–285)	0.85	878 (678–1079)	243 (236–871)	0.75
ALT peak, UI/L	133 (110–218)	137 (129–178)	0.47	1214 (713–1714)	663 (491–958)	0.61
Hospital stay, days	18 (13–19)	9 (8–19)	0.34	12 (11–14)	20 (14–25)	0.44
CD > 3°	0	0	1.0	0	1	1.0
CCI at discharge	20.9	8.7	0.37	8.7	33.5	0.23
Vascular complication	0 (0)	0 (0)	1.0	0 (0)	0 (0)	1.0
Biliary complications tot:	0 (0)	2 (67)	0.68	1 (33)	0 (0)	0.37
-ischemic cholangiopathy	0 (0)	1 (33)		1 (33)	0 (0)	
-anastomotic stenosis	0 (0)	1 (33)		0 (0)	0 (0)	
-fistula	0 (0)	0 (0)		0 (0)	0 (0)	
-stones	0 (0)	0 (0)		0 (0)	0 (0)	
Bil at 1 year, mg/dL	0.490 (0.465–0.495)	0.630 (0.615–1.1)	0.22	1.15 (1.07–1.17)	1.16 (0.99–1.33)	0.87
GGT at 1 year, UI/L	25 (19.5–27.2)	20 (18–52)	0.48	58 (46–99.5)	12 (6.5–17.5)	0.22
1-year graft survival %	100	100	1	100	67	0.37
1-year patient survival, %	100	100	1	100	100	1
Median follow-up, months	38 (37.5–38)	38 (37.5–38)	1	32 (27.5–32.5)	33 (30–35)	0.52

Abbreviations: SCS: static cold storage; NMP: normothermic machine perfusion; D-HOPE: dual hypothermic machine perfusion; BMI: body mass index; CVA: cerebrovascular accident; ICU: intensive care unit; IA: artery hypertension; DM: diabetes mellitus; MELD: model for end-stage liver disease; CIT: cold ischemia time; WIT: warm ischemia time; PNF: primary non-function; PRS: post-reperfusion syndrome; EAD; early allograft disfunction; AKI: acute kidney insufficiency; RRT: renal replace therapy; PRBC: packed red blood cell; FFP: fresh frozen plasma; CD: Clavien-Dindo; CCI: Comprehensive comorbidity index. Data are reported in median and interquartile range or number and percentage as appropriate. Bold characters in the table footer are used to denote the table’s subtitles.

**Table 3:** Perfusate cytokine concentration and mass balance.

Cytokine	Start	1 h	2 h	3 h	4 h
Overall Concentration *					
**D-HOPE**					
IL-1	2.0 (1.55–2.0)	2.05 (1.55–2.25)	2.15 (1.57–2.2)	\	\
IL-6	1.65 (1.2–3.75)	3.05 (2.02–5.05)	2.65 (2.05–5.65)	\	\
IL-10	2.35 (2.2–2.88)	3.1 (2.4–3.27)	3.15 (2.5–3.2)	\	\
TNF-a	0.3 (0.1–1.85)	0.1 (0.1–0.1)	0.1 (0.1–0.1)	\	\
**NMP**					
IL-1	0.1 (0.1–0.2)	4.4 (2.6–11.0)	10.8 (5.95–49.7)	15.3 (8.3–129)	153 (77.0–228)
IL-6	3.0 (1.55–4.4)	62.0 (190–20.5)	139 (389–154)	158 (115–402)	364 (219–509)
IL-10	0.1 (0.1–0.1)	61.9 (30.2–110)	48.3 (38.9–108)	40.8 (29.1–77.2)	17.9 (10.9–70.6)
TNF-a	0.1 (0.1–0.1)	23.1 (3.15–88)	18.8 (9.15–183)	19.1 (3.8–108)	7.3 (2.4–11.8)
**Mass Balance (pg) #**					
**D-HOPE**		**0–60 min**	**60–120 min**	**120–180 min**	**180–240 min**
IL-1		1.080 (0–1560)	1.440 (864–2.400)	\	\
IL-6		2.400 (660–7.380)	4.320 (0–11.880)	\	\
IL-10		0 (0–1.620)	0 (0–6.480)	\	\
TNF-a		0 (0–2.640)	0 (0–2.880)	\	\
**NMP**		**0–60 min**	**60–120 min**	**120–180 min**	**180–240 min**
IL-1		528 (0–801)	4.596 (0–8.184)	0 (0–108.768)	3.951 (0–87.837)
IL-6		2.112 (0–24.297)	229.215 (0–591.294)	289.677 (0–797.220)	271.302 (0–718.428)
IL-10		11.250 (8.316–63.279)	166.026 (123.192–319.176)	159.777 (61.893–160.680)	99.636 (39.852–104.043)
TNF-a		1.056 (0–87.042)	11.682 (0–706.893)	30.498 (0–678.564)	148.821 (22.356–517.845)

Abbreviations: NMP: normothermic machine perfusion; D-HOPE: dual hypothermic machine perfusion. * Data are expressed in median and interquartile range. # Data are expressed in median, as well as minimum and maximum values. Bold characters in the table footer are used to denote the table’s subtitles.

## Data Availability

No new data were created or analyzed in this study. Data sharing is not applicable due to privacy or ethical restrictions.

## Abbreviations

The following abbreviations are used in this manuscript:

ALT	alanine amino transferase
AT	aspartate amino transferase
AKI	acute kidney injury
BMI	body mass index
CCI	comprehensive complication index
CA	cytokine adsorption
CNT	national transplant authority
DBD	donation after brain death
D-HOPE	dual hypothermic oxygenated machine perfusion
EAD	early allograft dysfunction
fWIT	functional warm ischemia time
tWIT	total warm ischemia time
GGT	gamma glutamyl transpeptidase
IC	ischemic cholangiopathy
ICU	intensive care unit
IL-1	interleukin 1
IL-10	interleukin 10
IL-6	interleukin 6
IQR	interquartile range
KDIGO	kidney disease improving global outcomes
LT	liver transplantation
WIT	warm ischemia time
MEAF	model for early allograft function
MELD	model for end-stage liver disease
MP	machine perfusion
NMP	normothermic machine perfusion
NRP	normothermic regional perfusion
PNF	primary non-function
PRS	post-reperfusion syndrome
TNF-a	tumor necrosis factor

## Appendix B

**Table A1:** Histology.

	DHOPE w CA	DHOPE w/o CA	*p*	NMP w CA	NMP w/o CA	*p*
**Liver Parenchyma**						
**End of Procurement**						
-Fibrosis 1-2, n	2 (66)	3 (100)	0.374	2 (66)	2 (66)	0.495
-Necrosis > 10%, n	0	0	0	0	0	
-Macrosteatosi > 30%	0	0	0	0	0	
-Microsteatosis > 0%	0	0	0	0	0	
-PAS	60 (45–75)	90 (65–90)	0.609	85 (82.5–87.5)	80 (60–80)	0.374
-Suzuki’s score	1 (0.5–1)	1 (0.5–1)	1.0	1 (0.5–1.5)	1 (1–1.5)	0.724
0	1 (33)	1 (33)	0	1 (33)	0 (0)	
1	2 (66)	2 (66)	0	1 (33)	2 (66)	
2	0	0	0	1 (33)	1 (33)	
**End of MP**						
-Fibrosis 1-2, y	2 (66)	3 (100)	0.374	2 (66)	1 (33)	0.219
-Necrosis > 10%, y	0	0	0	1 (33)	1 (33)	0.789
-Macrosteatosi > 30%	0	0	0	0	0	
-Microsteatosi > 10%	0	0	0	0	0	
-PAS	80 (60–85)	80 (75–80)	0.692	75 (72.5–77.5)	70 (55–75)	0.518
-Suzuki’s score	1 (0.5–1)	0 (0–0.5)	0.519	1.5(1.25–1.75)	1 (1–2)	0.870
0	1 (33)	2 (66)	0	0	0	
1	2 (66)	1 (33)	0	1 (33)	2 (66)	
2	0	0	0	1 (33)	0	
3	0	0	0	0	1 (33)	
**End of LT**						
-Fibrosis 1-2, y	2 (66)	3 (100)	0.374	0	2 (66)	0.219
-Necrosis > 10%, y	0	0	0	1 (33)	3 (100)	0.272
-Macrosteatosi > 30%	0	0	0	0	1 (33)	0.495
-Microsteatosi > 10%	0	1 (33)	0.374	0	2 (66)	0.219
-PAS %, median (IQR)	42.3 (23.5–60)	50 (40–65)	0.690	30 (15–45)	70 (50–80)	0.373
-Suzuki’s score	0 (0–0.5)	1 (1–1)	0.116	1 (0.5–1.5)	3 (2.5–4.5)	0.219
0	2 (66)	0	0	1 (33)	0	
1	1 (33)	3 (100)	0	0	0	
2	0	0	0	1 (33)	1 (33)	
3	0	0	0	0	1 (33)	
6	0	0	0	0	1 (33)	
**Bile duct**						
**End of Procurement**						
-Epithelial damage	3 (100)	3 (100)		2 (66)	3 (100)	0.374
-Necrosis	0	0		0	0	
-Vascular plexus damage	0	0		0	0	
-Intravascular thrombi	0	0		0	0	
-Intramural bleeding	0	0		0	0	
-Super peribiliary gland damage	2 (66)	3 (100)	0.374	2 (66)	3 (100)	
-Deep peribiliary gland injury	1 (33)	0	0.374	1 (33)	0	0.272
-Inflammation	0	0		0	2 (66)	0.219
**End of MP**						
-Epithelial damage	3 (100)	3 (100)	1.0	2 (66)	3 (100)	0.374
-Necrosis	0	0		1 (33)	1 (33)	0.789
-Vascular plexus damage	0	0		0	0	
-Intravascular thrombi	0	0		0	0	
-Intramural bleeding	0	0		0	0	
-Super peribiliary gland damage	3 (100)	3 (100)	1.0	2 (66)	3 (100)	0.374
-Deep peribiliary gland injury	1 (33)	0	0.374	1 (33)	0	0.272
-Inflammation	0	0		1 (33)	3 (100)	0.272
**End of LT**						
-Epithelial damage	2 (66)	2 (66)	1.0	2 (66)	1 (33)	0.219
-Necrosis	0	0		2 (66)	1 (33)	0.219
-Vascular plexus damage	0	0		0	0	
-Intravascular thrombi	0	0		0	0	
-Intramural bleeding	0	0		0	0	
-Super peribiliary gland damage	2 (66)	2 (66)	1.0	1 (33)	2 (66)	0.219
-Deep peribiliary gland injury	0	1 (33)	0.423	0	0	0.667
-Inflammation	0	0		0	1 (33)	0.495

Bold characters in the table footer are used to denote the table’s subtitles.

**Table A2:** Bile characteristics stratified by perfusion technique and use of CA.

	D-HOPE w CA	D-HOPE w/o CA	NMP w CA	NMP w/o CA
**Day 1**				
-pH	7.73 ± 0.2	7.9 ± 0.1	7.87 ± 0.2	7.85 ± 0.2
-Lactate (mmol/L)	0.7 ± 0.4	0.65 ± 0.4	0.5 ± 0.0	4.8 ± 7.0
-HCO3	26.4 ± 2.6	28.5 ± 2.3	30.5 ± 1.2	22.7 ± 2.2
-glucose (mg/dL)	57 ± 59	12.5 ±5.4	15 ± 4.0	28 ± 23.5
-Na, mEq/L	140 ± 1.0	137 ± 2.1	140 ± 2.1	139 ± 6.5
-K, mEq/L	4.7 ± 0.6	4.7 ± 0.2	4.5 ± 0.3	4.4 ± 0.8
-Cl, mEq/L	114 ± 6.5	114 ± 5.0	114 ± 7.1	112 ± 2.9
-pO2	152 ± 12.5	169 ± 1.4	159 ± 9.6	174 ± 6.6
**Day 3**				
-pH	7.9 ± 0.1	8.0 ± 0.0	7.9 ± 0.2	8.0 ± 0.0
-Lactate (mmol/L)	0.6 ± 0.5	0.6 ± 0.1	0.5 ± 0.1	0.7 ± 0.6
-HCO_3_	35.6 ± 3.0	37.0 ± 0.0	33.8 ± 0.0	out of scale
-glucose (mg/dL)	13.5 ± 11.8	7.5 ± 3.7	8.0 ± 1.4	9.0 ± 7.0
-Na, mEq/L	143 ± 3.0	138 ± 1.4	141.7 ± 2.5	137.5 ± 2.1
-K, mEq/L	4.2 ± 0.3	4.2 ± 0.3	4.0 ± 0.2	4.2 ± 0.7
-Cl, mEq/L	111.5 ± 1.5	109 ± 7.1	106 ± 5.3	103 ± 7.1
-pO_2_	152.5 ± 15.5	165 ± 8.5	166.7 ± 28.4	176.0 ± 0.0
**Day 7**				
-pH	7.7 ± 0.1	7.7 ± 0.0	7.9 ± 0.2	7.7 ± 0.1
-Lactate (mmol/L)	0.9 ± 0.5	1.1 ± 0.4	0.9 ± 0.6	0.6 ± 0.4
-HCO_3_	26.3 ± 4.0	28.2 ± 8.0	29.8 ± 0.0	24.7 ± 1.4
-glucose (mg/dL)	10.3 ± 10.2	14.0 ± 14.1	10.0 ± 1.4	7.5 ± 4.9
-Na, mEq/L	138 ± 6.2	133 ± 4.2	135.5 ± 2.1	139 ± 9.9
-K, mEq/L	4.3 ± 0.2	4.7 ± 0.9	4.3 ± 0.0	4.1 ± 0.6
-Cl, mEq/L	105 ± 2.1	103 ± 1.4	106.5 ± 0.7	109 ± 3.5
-pO_2_	150 ± 10.8	152 ± 43.8	177 ± 8.4	157 ± 16.9

Abbreviations: D-HOPE: dual hypothermic oxygenated machine perfusion; w: with; CA: cytokine adsorption; w/o: without; NMP: normothermic machine perfusion. Data are expressed in mean ± SD.
